# Comparison between minimally invasive plate osteosynthesis and open reduction-internal fixation for proximal humeral fractures: a meta-analysis based on 1050 individuals

**DOI:** 10.1186/s12891-019-2936-y

**Published:** 2019-11-18

**Authors:** Feilong Li, Xuqiang Liu, Fuqiang Wang, Zhiping Gu, Qianyuan Tao, Cong Yao, Xuwen Luo, Tao Nie

**Affiliations:** Department of Orthopedics, the First Affiliated Hospital of Nanchang University, Nanchang, Jiangxi China

**Keywords:** Minimally invasive plate osteosynthesis (MIPO), Open reduction–internal fixation (ORIF), Proximal humeral fractures, Meta-analysis

## Abstract

**Background:**

This meta-analysis aimed to compare the clinical outcomes and complications of minimally invasive plate osteosynthesis (MIPO) and open reduction–internal fixation (ORIF) in patients with proximal humeral fractures.

**Methods:**

We searched PubMed, EMBASE, Ovid, and the Cochrane Library to identify all relevant studies from inception to April 2019. Cochrane Collaboration’s Review Manage 5.3 was used for meta-analysis.

**Results:**

Sixteen studies involving 1050 patients (464 patients in the MIPO group and 586 patients in the ORIF group) were finally included. According to the meta-analysis, MIPO was superior to ORIF in operation time, blood loss, postoperative pain, fracture union time, and constant score. However, MIPO was associated with more exposure to radiation and axillary nerve injury. No significant differences were found in length of hospital stays and complication except for axillary nerve injury.

**Conclusion:**

The present evidence indicates that compared to ORIF, MIPO had advantages in functional outcomes, operation time, blood loss, postoperative pain, and fracture union time for the treatment of PHFs. However, the MIPO technique had a higher rate of axillary nerve injury and longer radiation time compared to ORIF.

## Background

Fractures of the proximal humerus are the third most common osteoporotic fracture type [1], accounting for 4–5% of all fractures [2]. By 2030, the number of proximal humeral fractures (PHFs) will increase three times due to the increasing geriatric population [3]. Nondisplaced or minimally displaced PHFs can be successfully treated in a nonsurgical manner [4]. However, seriously displaced or unstable fractures usually require surgical treatment to achieve normal shoulder function [5]. There are many surgical strategies that were proven to be clinically effective, including minimally invasive plate osteosynthesis (MIPO), open reduction–internal fixation (ORIF), intramedullary nails, and primary arthroplasty [6]. Among those, ORIF with a locking plate is the commonly preferred surgical modality [7]; however, ORIF is associated with complications such as avascular necrosis of the humeral head and nonunion and infection due to extensive soft tissue stripping [8].

Recently, with the development of the concept of minimally invasive technologies and biological fixation, the MIPO has been widely used in the treatment for PHFs [9, 10]. MIPO via the deltoid-splitting approach minimizes soft tissue dissection, effectively reduces postoperative pain, and improves bone healing [11].

Although a meta-analysis has compared the clinical outcomes and complications of MIPO and ORIF for treatment PHFs [12], it only included seven studies, and more published data have become available in recent years. Therefore, we conducted a meta-analysis of all available comparative studies to compare the clinical outcomes and complications between MIPO and ORIF in the treatment of PHFs. Furthermore, we performed subgroup analysis of the constant score for a more comprehensive meta-analysis.

## Methods

### Aim

The objective of this meta-analysis was to compare clinical outcomes and complications of MIPO and ORIF in patients with PHFs.

### Search strategy

The meta-analysis was conducted in accordance with the PRISMA (Preferred Reporting Items for Systematic Reviews and Meta-Analyses) statements [13]. We searched PubMed, EMBASE, Ovid, and the Cochrane Library to identify all relevant studies from inception to April 2019. The search terms were “proximal humeral fracture,” “shoulder fractures,” “humerus surgical neck fracture,” “open reduction–internal fixation,” “ORIF,” “minimally invasive,” and “MIPO.” Additionally, the reference lists of relevant studies were manually searched. Languages were not restricted.

### Study selection

The studies that met the following inclusion criteria were selected: population (all PHFs), intervention (MIPO), control (ORIF), outcomes (blood loss, operative time, time of radiation exposure, fracture healing time, postoperative pain, function score, and complications), and study design (randomized [RCT] or nonrandomized control trial [non-RCT]). We excluded animal studies, case reports, letters, multiple publications, and patients with pathological fractures.

### Data extraction

Two reviewers (F.L.L. and F.Q.W.) independently extracted relevant data from the included studies. Discrepancies between data extracted were resolved by discussion between the two reviewers; if consensus was not reached, another author (T.N.) was consulted. The following data were extracted: the first author’s name, publication year, sample size, interventions, mean age, male/female ratio, duration of follow-up, fracture type, blood loss, operation time, duration of radiation exposure, postoperative pain, duration of fracture healing, functional outcomes, and complications.

### Quality assessment

Two reviews (F.L.L. and F.Q.W.) independently evaluated the methodological qualities and risk of bias of the non-RCTs with use of Methodological Index for Nonrandomized Studies (MINORS) [14]. The same two researchers assessed the quality of the RCTs using the *Cochrane Handbook*. A third reviewer resolved disagreements.

### Statistical analysis

All of the data were analyzed by Review Manager version 5.3 provided by the Cochrane Collaboration (London, UK). Continuous variables were expressed as mean differences (MDs) or standard mean differences (SMDs) and 95% confidence intervals (CIs). Dichotomous variables were presented as odds ratios (ORs) with 95% CI. A *P* value < 0.05 was considered statistically significant. The heterogeneity between studies was assessed by chi-square test and I^2^ test. If there was significant heterogeneity (*P* < 0.1 or I^2^ > 50%), a random-effects model was used for the meta-analysis. Otherwise, a fixed-effects model was used. Publication bias was evaluated by funnel plot.

## Results

### Literature search

**Fig. 1:**
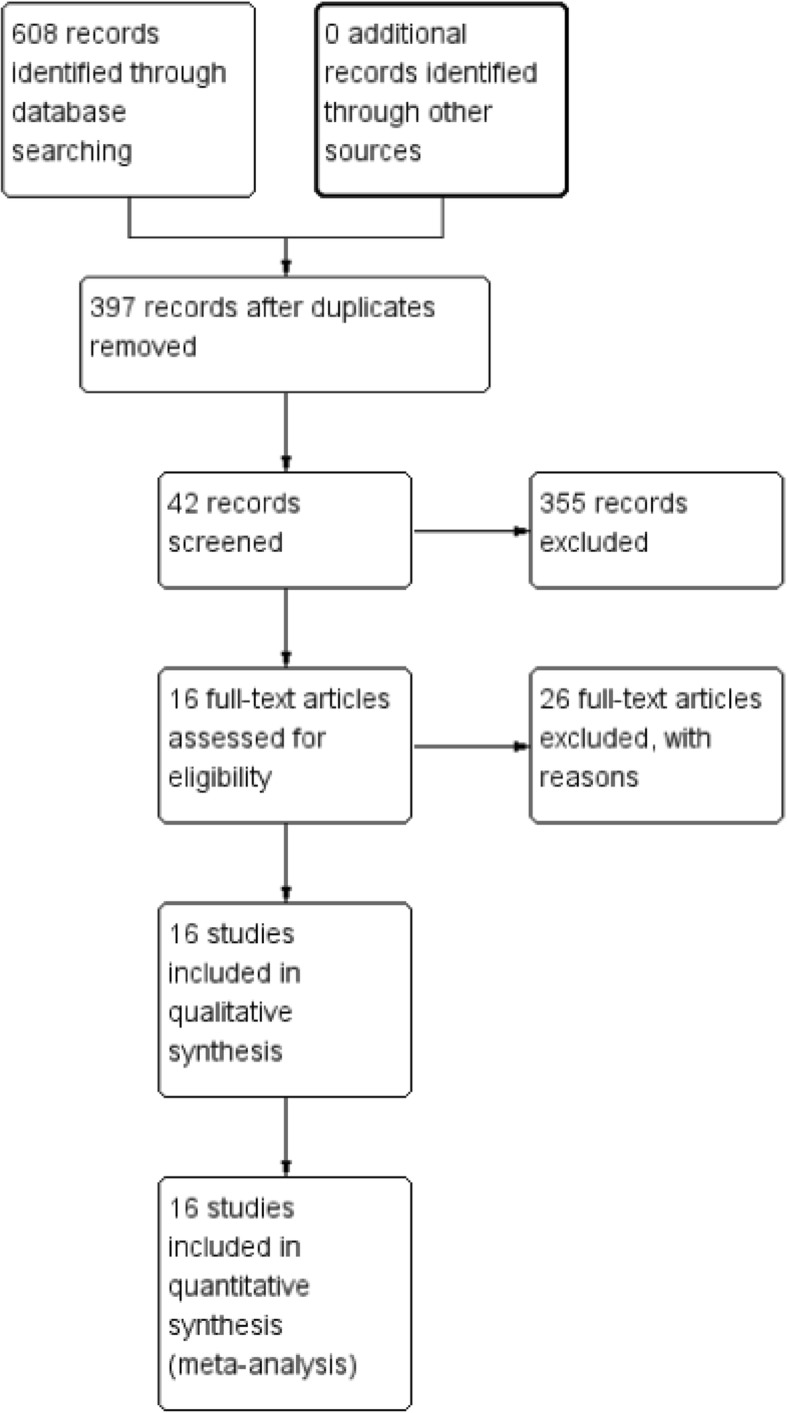
Flow diagram of studies processed for inclusion

A total of 608 potentially relevant studies were identified. The full search strategy for PubMed database is shown as Additional file 1. After removing 211 duplicates, we screened 397 papers. By reading the title and abstract, 355 papers were excluded according to the inclusion and exclusion criteria. A total of 42 studies were assessed by reading the full text; eventually, 16 studies involving 1050 patients (464 patients in the MIPO group and 586 patients in the ORIF group) were included in the meta-analysis [9, 10, 15–28]. The flow diagram of the included studies is shown in Fig. 1. The characteristics of the included studies are listed in Table 1.

**Table 1:** Characteristics of included studies

Included studies	Cases: MIPO/ORIF	Sex: male/female	Mean age (years): MIPO/ORIF	Follow-up (months)	Fracture type
Chiewchantanakit 2015 [21]	12/16	12/16	52/62	NS/NS	Neer: 2,3
Fischer 2016 [20]	30/30	16/34	57.6/60.6	22.8/20.7	AO: A,B,C
Gao 2015 [16]	21/18	17/22	70/72	18.1/18.1	Neer: 2,3
Kim 2019 [28]	19/17	NS/NS	58.7/52.6	24/24	Neer: 2
Lin 2014 [22]	43/43	28/58	63/61	12.6/13.1	AO: A,B,C
Liu 2013 [17]	47/51	43/55	72.8/49.9	18.1/18.1	Neer: 3,4
Liu 2015 [10]	39/52	42/49	60.2/61.7	24/24	Neer: 2,3,4
Liu 2016 [25]	33/42	28/47	50.3/52.1	14.2/14.2	Neer: 2,3
Liu 2019 [15]	45/72	44/73	62.2/60.1	NS/NS	Neer: 2,3,4
Röderer 2011 [26]	46/61	32/75	67.6/65	12/12	AO: A,B,C
Shang 2013 [19]	24/54	19/59	61.6/60	33.8/33.8	Neer: 2,3,4
Shen 2018 [23]	20/26	20/26	70.4/70.9	16.8/16.8	Neer: 2
Sohn 2017 [9]	45/45	NS/NS	61/62.6	14.3/15	Neer: 2,3,4
Wang 2012 [18]	20/20	14/26	69.6/69.7	NS/NS	Neer: 2,3
Zhang 2018 [24]	13/20	14/19	66.1/61.5	12.4/11.9	Neer: 3
Zhao 2017 [27]	17/19	21/15	64/64.3	10/10	Neer: 2,3,4

*MIPO* Minimally invasive plate osteosynthesis, *ORIF* Open reduction–internal fixation, *NS* Not stated

### Methodological quality

**Fig. 2:**
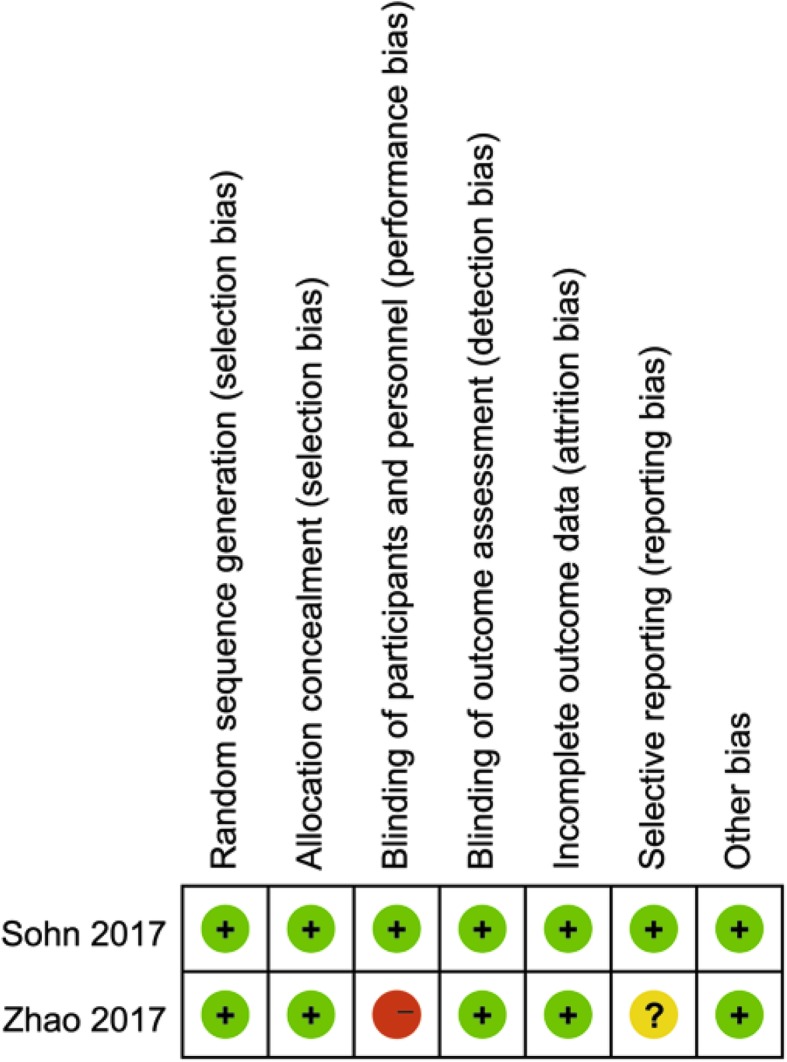
Risk of bias summary of all included randomized control trials. + represents yes; − represents no;? represents unclear

The methodological quality of the RCTs [9, 27] was assessed by the *Cochrane Handbook*, the assessment results are summarized in Fig. 2. The quality index scores of the non-RCTs [10, 15–26, 28] were 14–20. The assessment results are summarized in Table 2.

**Table 2:** MINORS appraisal scores for the included nonrandomized control trial

Name	Methodological items												Total
	1	2	3	4	5	6	7	8	9	10	11	12	
Chiewchantanakit 2015 [21]	2	2	0	2	0	2	2	0	2	2	2	2	18
Fischer 2016 [20]	2	2	2	2	0	2	2	0	2	2	2	2	20
Gao 2015 [16]	2	2	0	1	0	1	2	0	1	2	2	2	15
Kim 2019 [28]	2	2	0	2	0	2	2	0	2	0	2	2	16
Lin 2014 [22]	2	2	0	2	0	2	0	0	2	0	2	2	14
Liu 2013 [17]	2	2	0	1	0	2	2	0	2	2	2	2	17
Liu 2015 [10]	2	2	0	2	0	2	2	0	2	2	2	2	18
Liu 2016 [25]	2	2	0	2	0	2	2	0	2	2	2	2	18
Liu 2019 [15]	2	2	0	2	0	2	2	0	2	2	2	2	18
Röderer 2011 [26]	2	2	2	2	0	2	0	0	2	2	2	2	18
Shang 2013 [19]	2	2	0	2	0	2	0	0	2	2	2	2	16
Shen 2018 [23]	2	2	0	2	0	2	2	0	2	2	2	2	18
Wang 2012 [18]	2	2	0	1	0	1	2	0	2	2	2	2	16
Zhang 2018 [24]	2	2	0	2	2	2	2	0	2	2	2	2	20

*MINORS* Methodological index for nonrandomized studies

(1) A clearly stated aim; (2) inclusion of consecutive patients; (3) prospective collection of data; (4) endpoints appropriate to the aim of the study; (5) unbiased assessment of the study endpoint; (6) follow-up period appropriate to the aim of the study; (7) loss to follow-up that is < 5%; (8) prospective calculation of the study size; (9) an adequate control group; (10) contemporary groups; (11) baseline equivalence of groups; (12) adequate statistical analyses. The items were scored as “0” (not reported), “1” (reported but inadequate), or “2” (reported and adequate)

### Results of the meta-analysis

#### Blood loss

**Fig. 3:**
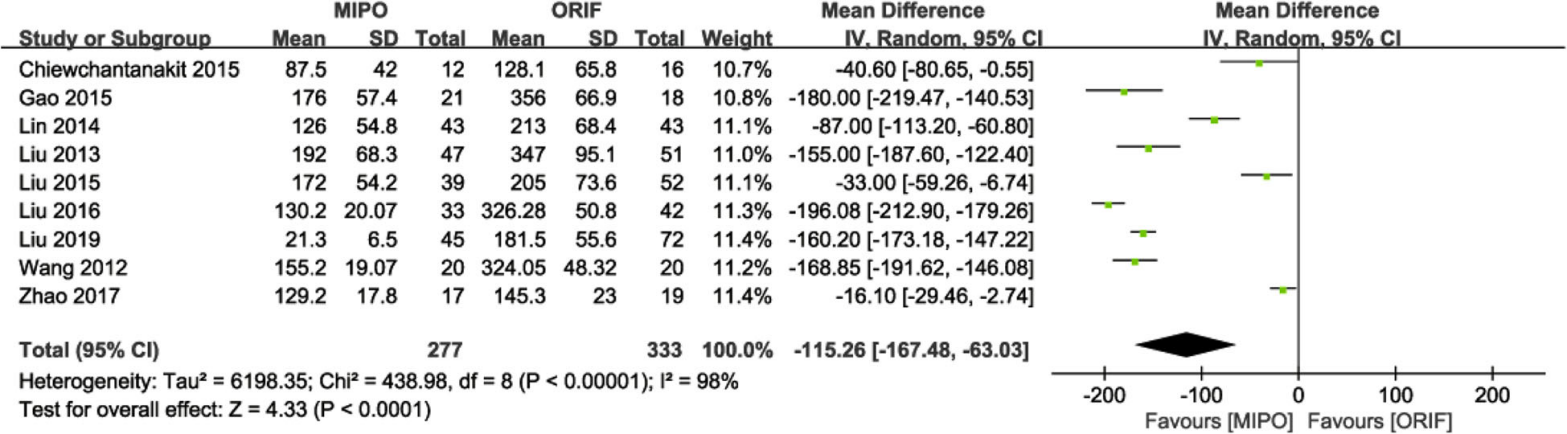
Forest plot for blood loss between the MIPO and ORIF groups. MIPO: minimally invasive plate osteosynthesis; ORIF: open reduction–internal fixation; OR: odds ratio; CI: confidence interval

Nine studies involving 610 patients reported blood loss [10, 15–18, 21, 22, 25, 27]. Heterogeneity tests indicated high heterogeneity (*P* < 0.00001; I^2^ = 98%); a random-effects model was used. The result showed lesser blood loss in the MIPO group than that in the ORIF group (MD = − 115.26; 95% CI: − 167.48 to − 63.03; *P* < 0.0001; Fig. 3).

#### Operation time

**Fig. 4:**
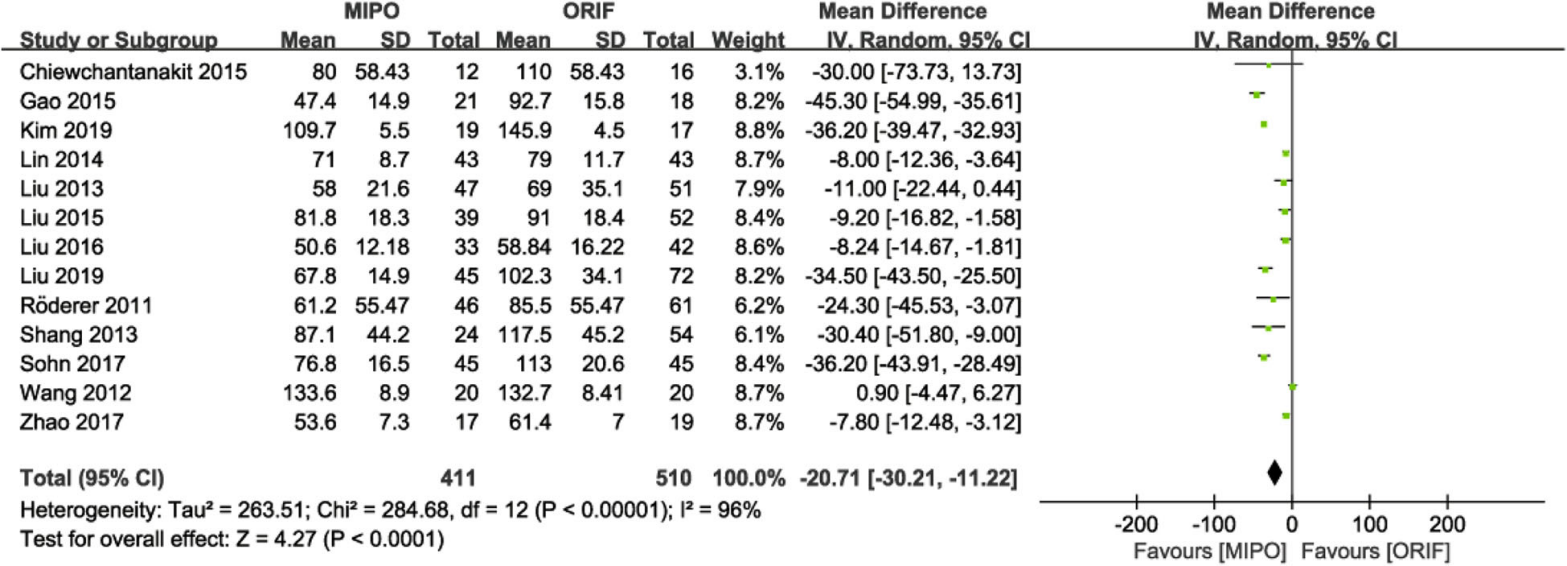
Forest plot for operation time between the MIPO and ORIF groups. MIPO: minimally invasive plate osteosynthesis; ORIF: open reduction–internal fixation; OR: odds ratio; CI: confidence interval

Thirteen studies [9, 10, 15–19, 21, 22, 25–28], with 921 patients, mentioned operation time, and the heterogeneity test indicated significant heterogeneity (*P* < 0.00001; I^2^ = 96%); thus, a random-effects model was adopted. The results showed shorter operation time in the MIPO group than that in the ORIF group (MD = − 20.71; 95% CI: − 30.21 to − 11.22; *P* < 0.0001; Fig. 4).

#### Radiation time

**Fig. 5:**
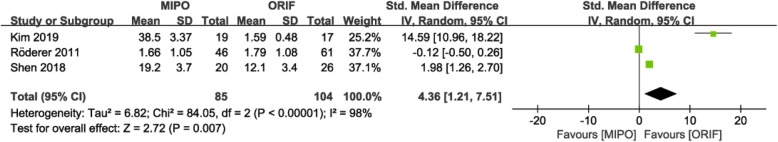
Forest plot for radiation time between the MIPO and ORIF groups. MIPO: minimally invasive plate osteosynthesis; ORIF: open reduction–internal fixation; OR: odds ratio; CI: confidence interval

Three articles [23, 26, 28], with 189 patients, stated radiation time, but used different units of time; therefore, the SMD was adopted. A random-effects model was used, with obvious heterogeneity (P < 0.00001; I^2^ = 98%). The duration of radiation exposure in the MIPO group was longer than that in the ORIF group (MD = 4.36; 95% CI: 1.21 to 7.51; *P* = 0.007; Fig. 5).

#### Postoperative pain

**Fig. 6:**
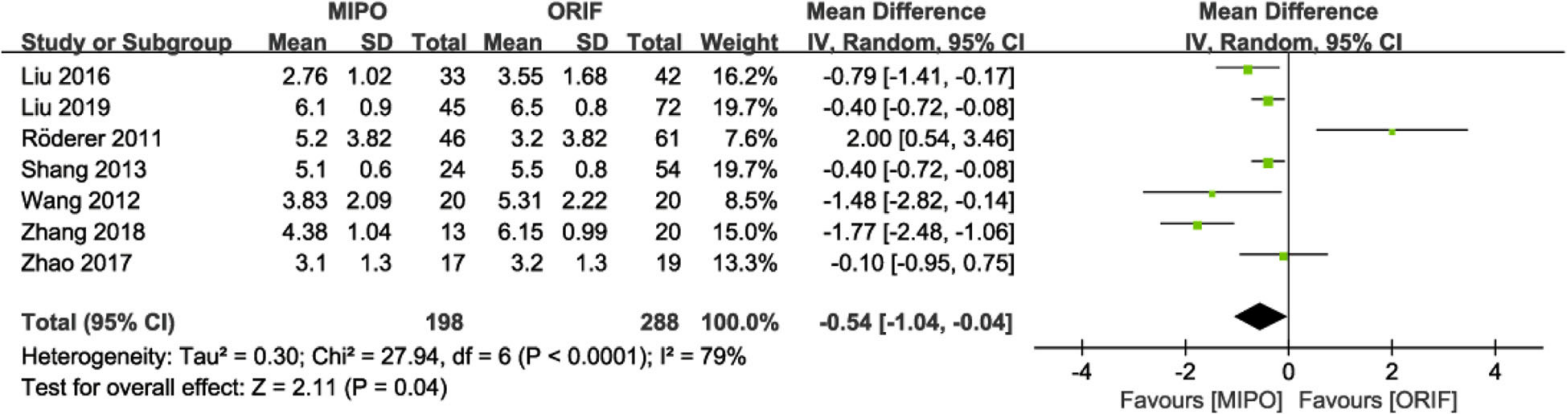
Forest plot for postoperative pain between the MIPO and ORIF groups. MIPO: minimally invasive plate osteosynthesis; ORIF: open reduction–internal fixation; OR: odds ratio; CI: confidence interval

The visual analogue scale (VAS) was used to evaluate postoperative pain, and seven studies [15, 18, 19, 24–27], with 486 patients, reported the VAS score. A random-effects model was used, with obvious heterogeneity (P < 0.0001; I^2^ = 79%). The meta-analysis showed a significantly lower VAS score in the MIPO group than in the ORIF group (MD = − 0.54; 95% CI: − 1.04 to − 0.04; *P* = 0.04; Fig. 6).

#### Union time

**Fig. 7:**
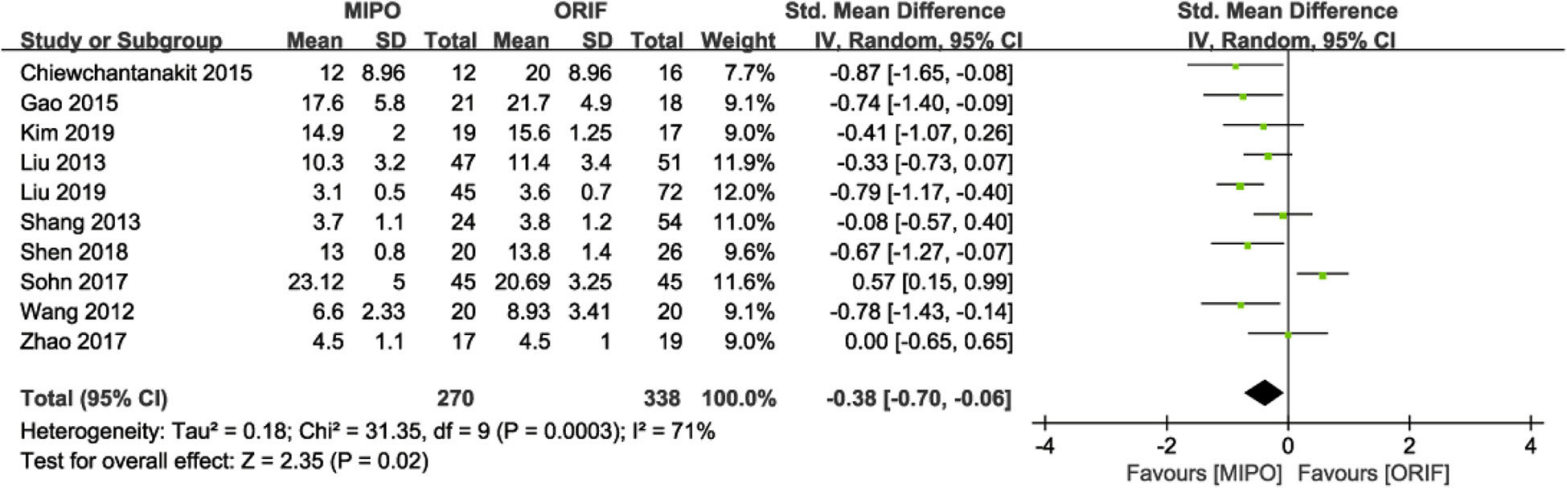
Forest plot for union time between the MIPO and ORIF groups. MIPO: minimally invasive plate osteosynthesis; ORIF: open reduction–internal fixation; OR: odds ratio; CI: confidence interval

Ten studies [9, 15–19, 21, 23, 27, 28], with 608 patients, indicated postoperative union time, but used different units of time; therefore, the SMD was adopted. Heterogeneity tests indicated that significant heterogeneity (*P* = 0.0003; I^2^ = 71%); thus, a random-effects model was adopted. The meta-analysis showed a shorter time to union in the MIPO group than in the ORIF group (SMD = − 0.38; 95% CI: − 0.70 to − 0.06; *P* = 0.02; Fig. 7).

#### Functional outcomes

**Fig. 8:**
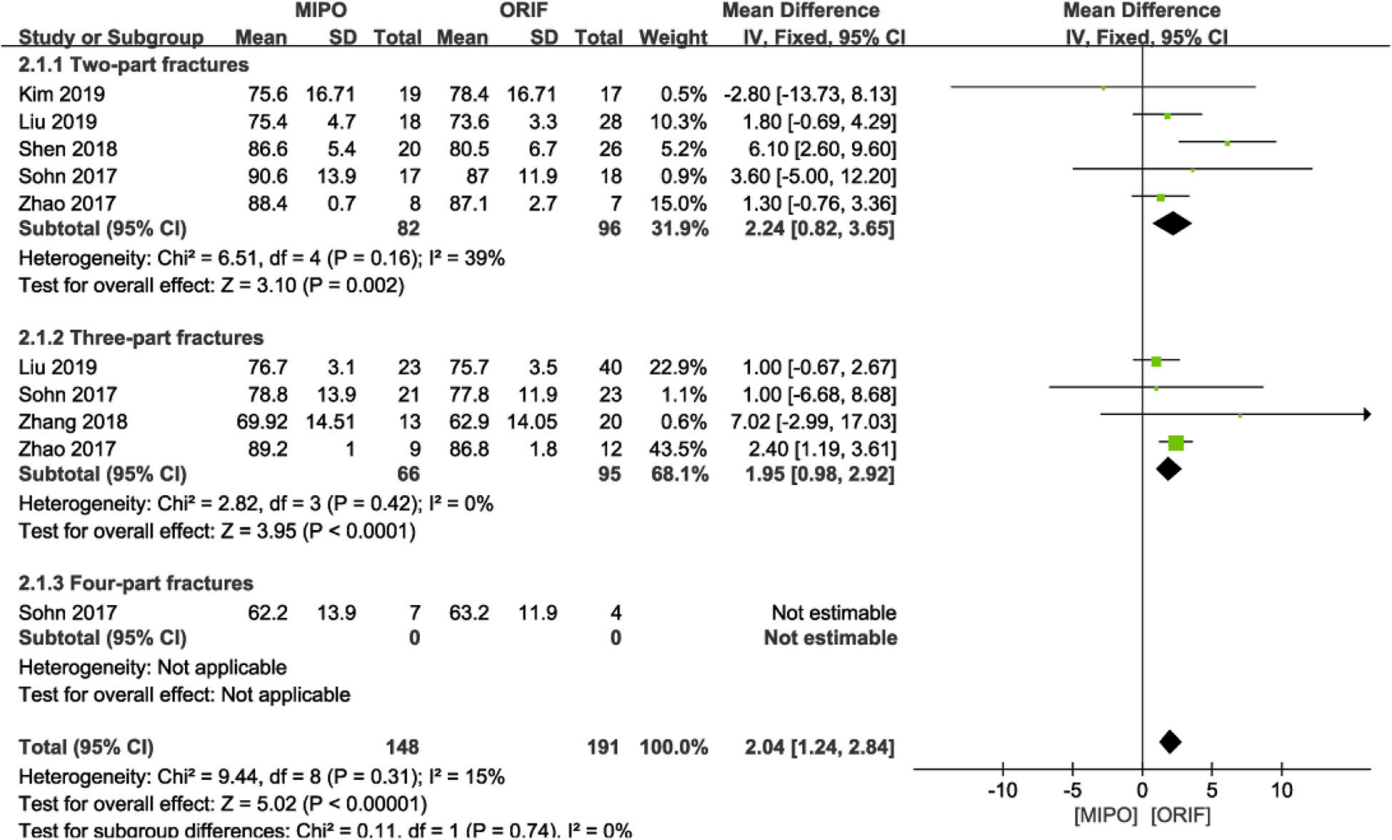
Forest plot for constant score between the MIPO and ORIF groups. MIPO: minimally invasive plate osteosynthesis; ORIF: open reduction–internal fixation; OR: odds ratio; CI: confidence interval

The constant score of Neer type II fractures were provided in four studies [9, 15, 23, 27, 28]. A fixed-effects model was used (*P* = 0.16; I^2^ = 39%), and analysis showed significantly higher score in the MIPO group than in the ORIF group (MD = 2.24; 95% CI: 0.82 to 3.65; P = 0.02; Fig. 8).

The constant score of Neer type III fractures were stated in four studies [9, 15, 24, 27]. A fixed-effects model was used (*P* = 0.42; I^2^ = 0%), and analysis showed significantly higher score in the MIPO group than in the ORIF group (MD = 1.95; 95% CI: 0.98 to 2.92; *P* < 0.001; Fig. 8).

Meta-analysis was not performed for the constant score of Neer type IV PHFs because it was reported by only one study.

#### Axillary nerve injury

Four studies [17, 22, 23, 25], with 305 patients, reported axillary nerve injury. A fixed-effects model was used (*P* = 0.95; I^2^ = 0%), and results showed a significantly higher rate of axillary nerve injury in the MIPO group than in the ORIF group (OR = 4.88; 95% CI: 1.03 to 23.25; *P* = 0.05).

#### Complications

**Fig. 9:**
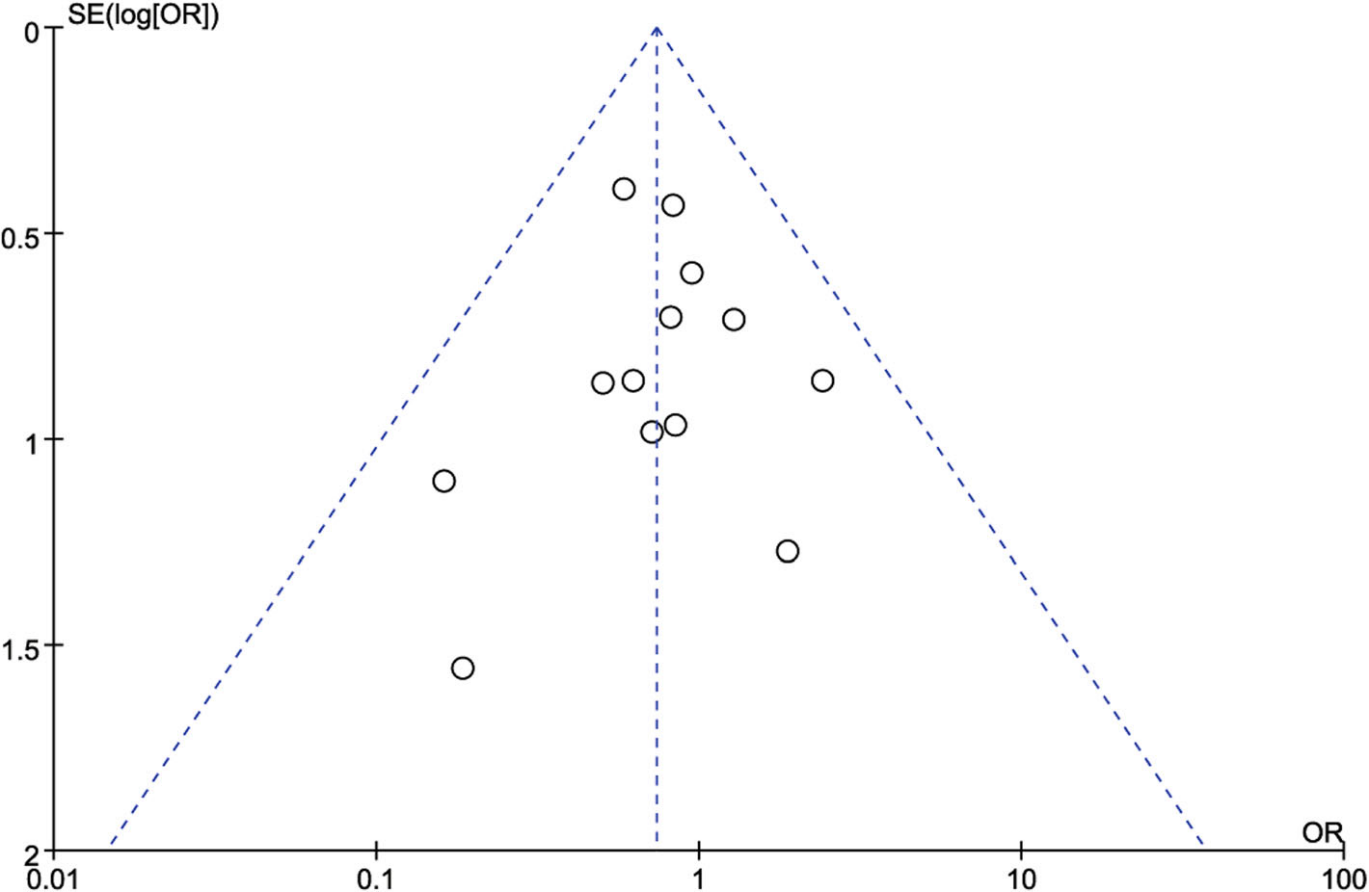
Funnel plot for publication bias. OR: odds ratio, SE: standard error

Thirteen studies reported complications. A fixed-effects model was used (*P* = 0.88; I^2^ = 0%), and pooled results showed no significant difference in total complication rate between the two groups (OR = 0.74; 95% CI: 0.51 to 1.07; *P* = 0.11; Fig. 9). However, the MIPO group had a significantly higher rate of axillary nerve injury that the ORIF group (OR = 4.88; 95% CI: 1.03 to 23.25; P = 0.05; I^2^ = 0%). The pooled results of the following complications showed no significant difference between the two groups (Table 3): avascular necrosis, impingement, screw perforation, implant loosening, delayed union or nonunion, limited abduction, and varus.

**Table 3:** Meta-analysis of reported complications

Outcomes	No. of trials	No. of patients: MIPO/ORIF	OR (95% CI)	*P* value	I^2^ (%)	*P* value for heterogeneity
Nerve injury	4	143/162	4.88 (1.03, 23.2)	0.05	0	0.95
Impingement	4	154/179	0.96 (0.36,2.54)	0.94	0	0.94
Screw perforation	3	134/149	0.97 (0.42,2.23)	0.94	0	0.65
Implant loosening	6	222/281	0.70 (0.28,1.75)	0.44	24	0.25
Avascular necrosis	7	251/299	0.41 (0.16,1.05)	0.06	0	0.78
Delayed union or nonunion	6	207/307	0.37 (0.12,1.13)	0.08	0	0.97
Limited abduction	3	108/178	0.73 (0.17,3,26)	0.69	0	0.58
Varus	5	140/155	1.35 (0.47,3.90)	0.58	0	0.62

*MIPO* Minimally invasive plate osteosynthesis, *ORIF* Open reduction–internal fixation, *OR* Odds ratio, *CI* Confidence interval

#### Publication bias

**Fig. 10:**
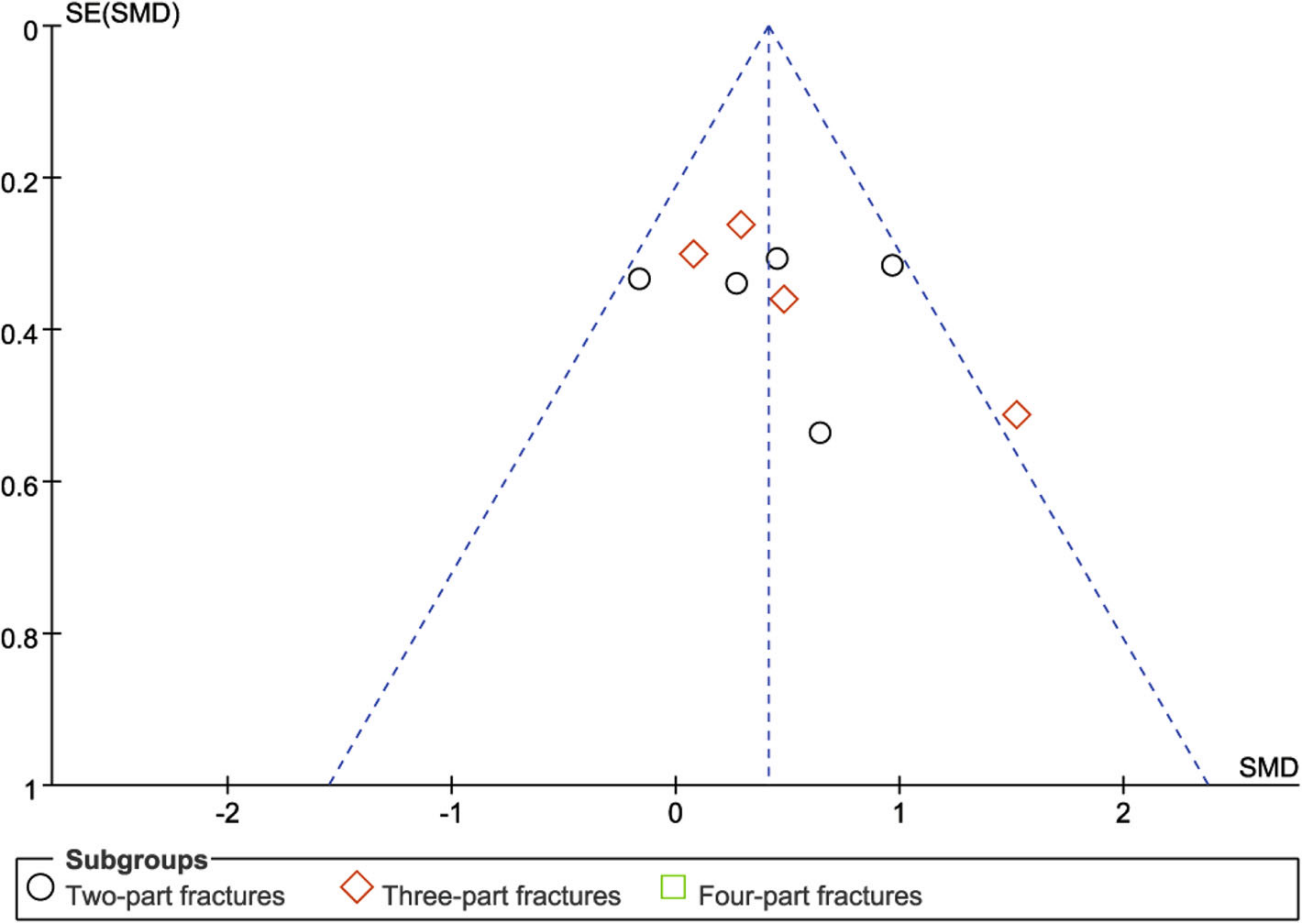
Funnel plot for publication bias. SE: standard error, SMD: standard mean difference

Funnel plots of the total complication rate (Fig. 9), and functional outcomes (Fig. 10) showed no substantial asymmetry, indicating no significant risk for publication bias.

#### Sensitivity analysis

After sensitivity analysis, operation time and union time became insignificant for randomized trials. This change may be due to the inclusion of only 2 randomized controlled trials. Results for all other outcomes remained unchanged (Table 4).

**Table 4:** Sensitivity analyses

Outcomes	Results Primary Analysis	RCT	non-RCT
Blood loss	−115.26(−167.48 to −63.03)	*	− 128.34(− 168.26 to −88.42)
Operation time	−20.71(−30.21 to −11.22)	−21.83(−49.66 to 6.00)	−20.56(−31.55 to −9.57)
Radiation time	4.36 (1.21 to 7.51)	*	4.36 (1.21 to 7.51)
Postoperative pain	−0.54(−1.04 to −0.04)	*	−0.60(− 1.16 to −0.04)
Union time	−0.38(−0.70 to −0.06)	0.34(−0.21 to 0.89)	−0.54(−0.75 to −0.34)
Functional outcomes	0.40 (0.18 to 0.61)	0.38 (0.01 to 0.74)	0.41 (0.13 to 0.68)
Axillary nerve injury	4.88 (1.03 to 23.25)	*	4.88 (1.03 to 23.25)
Complications	0.74 (0.51 to 1.07)	0.81 (0.37 to 1.76)	0.72 (0.47 to 1.09)

*: Analysis not performed because there was≤1 comparative study

## Discussion

We compared the clinical outcomes and complications of MIPO and ORIF in patients with PHFs in this meta-analysis. In contrast to a previously published meta-analysis [12], our meta-analysis involved a larger number of studies, but did not completely yield the same results. The findings of this study suggested that MIPO had advantages in operation time, blood loss, postoperative pain, fracture union time, and constant score compared with ORIF. However, MIPO had a higher rate of axillary nerve injury and longer radiation time compared with ORIF. There was no significant difference in complications between the two groups.

Over the past decade, the MIPO technique has become a more popular treatment for PHF [29]. Kim et al. [28] reported that the MIPO technique via the deltoid-splitting approach can provide sufficient field of vision of the plate location by minimal soft tissue dissection. Thus, it is easy to perform a reduction of a large greater tuberosity fragment [22], significantly reducing operation time and blood loss.

The MIPO technique minimizes incision and avoids damage to the deltoid muscle, which will reduce postoperative pain and facilitate early functional training [27]. Early functional training plays a positive role for recovery of shoulder joint function.

In this meta-analysis, the MIPO group had a significantly longer radiation duration because the patients underwent indirect reduction under fluoroscopy [28]. MIPO’s longer radiation time, compared with that of ORIF, is a negative aspect of MIPO.

Restoration to normal shoulder function is an important goal of the treatment of PHF. The present study showed that MIPO provides a better constant score of Neer type II or III PHFs. The result was similar to that of previous studies [23, 27]. Therefore, MIPO achieves better shoulder function in the treatment of PHFs.

Another important finding of this meta-analysis was the higher rate of axillary nerve injury in the MIPO group than in the ORIF group. Acklin et al. [30] reported that axillary nerve injury is the risk factor of the MIPO. However, Koljonen et al. [31] reported no axillary nerve injury in patients treated with MIPO. Whether axillary nerve lesions are more frequent in the MIPO approach remains controversial. Axillary nerve injury in the MIPO group may be related to the incisions in the deltoid-splitting approach extending more than 5 cm distal to the tip of the acromion [32]. To prevent injury to the axillary nerve with the MIPO technique, incisions should not extend more than 5 cm distal to the tip of the acromion [32]. In addition, the axillary nerve should be identified and protected by positioning the index finger on the nerve during the insertion of the plate on the proximal humerus [33].

Meta-analysis results indicated that MIPO had shorter time to union compared with that ORIF in PHFs. Similar results were also reported by five of the included studies [15, 16, 18, 21, 23]. The MIPO technique is commonly believed to provide advantages of fracture union process, as it maintains the periosteum and soft tissue around the fracture site [28].

The meta-analysis results showed no significant difference in impingement, screw perforation, implant loosening, avascular necrosis, delayed union or nonunion, limited abduction, and varus collapse between the MIPO and ORIF groups.

Our study has some limitations. First, the outcomes, except for the constant score, were not analyzed separately according to Neer classification. The main reason was that most studies did not show the data of interest in a separate form. Second, only two RCTs were included. Finally, the follow-up duration was short; longer follow-up may identify more complications. Therefore, RCTs with longer follow-up duration and larger number of samples are needed to confirm our results.

## Conclusion

The meta-analysis results showed that in comparison with ORIF, MIPO had advantages in operation time, blood loss, postoperative pain, and fracture union time for the treatment of PHFs. The MIPO technique was associated with better shoulder function in Neer type II or III PHFs. However, the MIPO technique had a higher rate of axillary nerve injury and longer radiation time compared to ORIF. There was no significant difference in complication rates between MIPO and ORIF. Recently, a network meta-analysis demonstrated that non-surgical treatment (NST) was associated with lower adverse event rates compared to ORIF for 3- and 4-part PHFs [34]. We recommend that future studies should not only compare MIPO to ORIF but also to NST to obtain thorough evidence-based treatment guidelines.

## Supplementary information

**Additional file 1.** Full search strategy for Pubmed database.

## Data Availability

All data generated or analysed during this study are included in this published article and its supplementary information files.

## Abbreviations

CI: Confidence intervals
MD: Mean difference
MINORS: Methodological Index for Nonrandomized Studies
MIPO: Minimally invasive plate osteosynthesis
OR: Odds ratio
ORIF: Open reduction–internal fixation
PHF: Proximal humeral fracture
PRISMA: Preferred Reporting Items for Systematic Reviews and Meta-Analyses
RCT: Randomized control trial
SMD: Standard mean difference
VAS: Visual analogue scale

## References

[1] Calvo E, Morcillo D, Foruria AM, Redondo-Santamaría E, Osorio-Picorne F, Caeiro JR (2011). Nondisplaced proximal humeral fractures: high incidence among outpatient-treated osteoporotic fractures and severe impact on upper extremity function and patient subjective health perception. J Shoulder Elbow Surg.

[2] Horak J, Nilsson BE (1975). Epidemiology of fracture of the upper end of the humerus. Clin Orthop Relat Res.

[3] Kannus P, Palvanen M, Niemi S, Parkkari J, Järvinen M, Vuori I (2000). Osteoporotic fractures of the proximal humerus in elderly Finnish persons: sharp increase in 1970-1998 and alarming projections for the new millennium. Acta Orthop Scand.

[4] Gaebler C, McQueen MM, Court-Brown CM (2003). Minimally displaced proximal humeral fractures: epidemiology and outcome in 507 cases. Acta Orthop Scand.

[5] Pinkas D, Wanich TS, DePalma AA, Gruson KI (2014). Management of malunion of the proximal humerus: current concepts. J Am Acad Orthop Surg.

[6] Maier D, Jaeger M, Izadpanah K, Strohm PC, Suedkamp NP (2014). Proximal humeral fracture treatment in adults. J Bone Joint Surg Am.

[7] Hirschmann MT, Fallegger B, Amsler F, Regazzoni P, Gross T (2011). Clinical longer-term results after internal fixation of proximal humerus fractures with a locking compression plate (PHILOS). J Orthop Trauma.

[8] Brunner F, Sommer C, Bahrs C, Heuwinkel R, Hafner C, Rillmann P (2009). Open reduction and internal fixation of proximal humerus fractures using a proximal humeral locked plate: a prospective multicenter analysis. J Orthop Trauma.

[9] Sohn HS, Jeon YS, Lee J, Shin SJ (2017). Clinical comparison between open plating and minimally invasive plate osteosynthesis for displaced proximal humeral fractures: a prospective randomized controlled trial. Injury..

[10] Liu K, Liu P-c, Liu R, Wu X (2015). Advantage of minimally invasive lateral approach relative to conventional deltopectoral approach for treatment of proximal humerus fractures. Med Sci Monit.

[11] Acklin YP, Sommer C (2012). Plate fixation of proximal humerus fractures using the minimally invasive anterolateral delta split approach. Oper Orthop Traumatol.

[12] Zang JC, Du JJ, Li C, Wang JB, Ma XL (2018). Comparison between minimally invasive plate osteosynthesis and open plating for proximal humeral fractures: a meta-analysis. J Comp Eff Res.

[13] Moher D, Liberati A, Tetzlaff J, Altman DG (2009). Preferred reporting items for systematic reviews and meta-analyses: the PRISMA statement. BMJ..

[14] Slim K, Nini E, Forestier D, Kwiatkowski F, Panis Y, Chipponi J (2003). Methodological index for non-randomized studies (minors): development and validation of a new instrument. ANZ J Surg.

[15] Liu BC, Yang ZW, Zhou F, Ji HQ, Zhang ZS, Guo Y (2019). Application of the modified internal fixation method of minimally invasive percutaneous plate osteosynthesis in treatment of proximal humeral fracture. Beijing Da Xue Xue Bao.

[16] Gao YB, Tong SL, Yu JH, Lu WJ (2015). Case control study on open reduction internal fixation (ORIF) and minimally invasive percutaneous plate osteosynthesis (MIPPO) for the treatment of proximal humerus fractures in aged. Zhongguo Gu Shang.

[17] Liu J, Li SH, Li ZH, Wang JG, Yang CX, Zhang L (2013). Case-control study on minimally invasive percutaneous new plate osteosynthesis applied in proximal humerus fractures in elder patients. Zhongguo Gu Shang.

[18] Wang JF, Song HB, Gu HJ, Ling ZD, Ma HH (2012). Case-control study on minimally invasive plate osteosynthesis for the treatment of proximal humeurs fractures in elderly patients. Zhongguo Gu Shang.

[19] Shang LP, Zhou F, Ji HQ, Zhang ZS, Liu XG, Tian Y (2013). Comparison of curative effects between minimally invasive locking plate internal fixation and open reduction with internal fixation for the treatment of proximal humerus fractures. Beijing Da Xue Xue Bao.

[20] Fischer C, Frank M, Kunz P, Tanner M, Weber MA, Moghaddam A (2016). Dynamic contrast-enhanced ultrasound (CEUS) after open and minimally invasive locked plating of proximal humerus fractures. Injury..

[21] Chiewchantanakit S, Tangsripong P (2015). Locking plate fixation of proximal humeral fracture: minimally invasive vs. standard delto-pectoral approach. J Med Assoc Thai.

[22] Lin T, Xiao B, Ma X, Fu D, Yang S (2014). Minimally invasive plate osteosynthesis with a locking compression plate is superior to open reduction and internal fixation in the management of the proximal humerus fractures. BMC Musculoskelet Disord.

[23] Shen QF, Wen X, Yang SW, Chen X, Fan WX, Xu GZ (2018). MIPPO and ORIF for the treatment of elderly proximal humerus fractures of type Neer II:a case control study. Zhongguo Gu Shang.

[24] Zhang Z, Zhang G, Peng Y, Wang X, Guo H, Zhang W (2018). Modified minimally invasive approach and intra-osseous portal for three-part proximal humeral fractures: a comparative study. J Orthop Surg Res.

[25] Liu YW, Wei XE, Kuang Y, Zheng YX, Gu XF, Zhan HS (2016). Open vs. closed reduction combined with minimally invasive plate osteosynthesis in humeral fractures. Minim Invasive Ther Allied Technol.

[26] Röderer G, Erhardt J, Kuster M, Vegt P, Bahrs C, Kinz L (2011). Second generation locked plating of proximal humerus fractures--a prospective multicentre observational study. Int Orthop.

[27] Zhao L, Yang P, Zhu L, Chen AM (2017). Minimal invasive percutaneous plate osteosynthesis (MIPPO) through deltoid-pectoralis approach for the treatment of elderly proximal humeral fractures. BMC Musculoskelet Disord.

[28] Kim YG, Park KH, Kim JW, Oh JK, Yoon JP, Kim HJ (2019). Is minimally invasive plate osteosynthesis superior to open plating for fixation of two-part fracture of the proximal humerus?. J Orthop Surg (Hong Kong).

[29] Gonç U, Atabek M, Teker K, Tanrıöver A (2017). Minimally invasive plate osteosynthesis with PHILOS plate for proximal humerus fractures. Acta Orthop Traumatol Turc.

[30] Acklin YP, Jenni R, Walliser M, Sommer C (2009). Minimal invasive PHILOS®- plate osteosynthesis in proximal humeral fractures. Eur J Trauma Emerg S.

[31] Koljonen PA, Fang C, Lau TW, Leung F, Cheung NWK (2015). Minimally invasive plate osteosynthesis for proximal humeral fractures. J Orthop Surg (Hong Kong).

[32] Ruchholtz S, Hauk C, Lewan U, Franz D, Kühne C, Zettl R (2011). Minimally invasive polyaxial locking plate fixation of proximal humeral fractures: a prospective study. J Trauma.

[33] Laflamme GY, Rouleau DM, Berry GK, Beaumont PH, Reindl R, Harvey EJ (2008). Percutaneous humeral plating of fractures of the proximal humerus: results of a prospective multicenter clinical trial. J Orthop Trauma.

[34] Orman S, Mohamadi A, Serino J, Murphy J, Hanna P, Weaver MJ, et al. Comparison of surgical and non-surgical treatments for 3- and 4-part proximal humerus fractures: a network meta-analysis. Shoulder Elbow. 2019. 10.1177/1758573219831506. 10.1177/1758573219831506 PMC7153210 32313559

