# Supplementary material for: Comparison between minimally invasive plate osteosynthesis and open reduction-internal fixation for proximal humeral fractures: a meta-analysis based on 1050 individuals
Source: BMC Musculoskelet Disord. 2019 Nov 18;20:550. doi: 10.1186/s12891-019-2936-y (PMC6862799; doi:10.1186/s12891-019-2936-y)
Supplement: Supplementary file 1 — Additional file 1. Full search strategy for Pubmed database. [file 12891_2019_2936_MOESM1_ESM.docx]

| Full search strategy for Pubmed database | | | |
| --- | --- | --- | --- |
| Database | Search string | Qualifiers/Filters | Results |
| Pubmed | (((shoulder fractures OR proximal humeral fracture OR proximal humeral fractures OR proximal humerus fracture OR proximal humerus fractures OR proximal humerus OR humerus surgical neck fracture OR humerus surgical neck fractures OR humeral surgical neck fracture OR humeral surgical neck fractures)) AND ((minimally invasive surgical procedures OR surgical procedures, minimal OR surgical procedures, minimal access OR surgical procedures, minimally invasive OR minimal access surgical procedures OR minimal surgical procedure OR procedures, minimally invasive surgical OR minimally invasive surgery OR minimally invasive surgeries OR surgeries, minimally invasive OR surgery, minimally invasive OR procedure, minimal surgical OR procedures, minimal access surgical OR procedures, minimal surgical OR surgical procedure, minimal OR minimal surgical procedures OR minimally invasive OR MIPO))) AND ((open fracture reduction OR fracture reduction, open OR open fracture reductions OR open reduction, fracture OR fracture open reduction OR fracture open reductions OR open reductions, fracture OR reduction, fracture open OR reductions, fracture open OR ORIF OR open)) | Publication date: to 20-April-2019 | 244 |
